# Adenocarcinoma with Enteroblastic Differentiation and Neuroendocrine Features in Autoimmune Gastritis: Novel Insights into Tumor Development from Endocrine Cell Micronests

**DOI:** 10.70352/scrj.lte.25-0017

**Published:** 2025-03-28

**Authors:** Hidetoshi Satomi, Shingo Ishiguro, Sei Murayama, Yoshiaki Andoh, Noriya Uedo, Tomoki Michida, Ryu Ishihara, Keiichiro Honma

**Affiliations:** 1 Department of Diagnostic Pathology and Cytology, Osaka International Cancer Institute, Osaka, Osaka, Japan; 2 PCL Osaka Pathology & Cytology Center, Osaka, Osaka, Japan; 3 Department of Gastrointestinal Oncology, Osaka International Cancer Institute, Osaka, Osaka, Japan

**Keywords:** autoimmune gastritis, adenocarcinoma with enteroblastic differentiation, neuroendocrine features, endocrine cell micronest, endoscopic resection

**To the Editor,**

Autoimmune gastritis leads to fundic gland atrophy and compensatory endocrine cell hyperplasia.^1)^ Enterochromaffin-like (ECL) cell proliferation induces the formation of endocrine cell micronests, with nodular aggregates ≥500 *μ*m considered neoplastic.^2)^ Here, we present a rare case of adenocarcinoma with enteroblastic differentiation (ACED) exhibiting neuroendocrine features in the context of autoimmune gastritis. Despite the poorly differentiated histology, which typically necessitates surgical resection, careful endoscopic assessment and precise technique achieved complete resection with negative margins.

A 60-year-old woman underwent endoscopic mucosal resection of a 10-mm polypoid lesion (0–Is) from the gastric corpus. This patient had no remarkable history of autoimmune diseases; the serum anti-Helicobacter pylori antibody titer was <3 U/mL (reference range, <10 U/mL). She had white light endoscopic findings characteristic of autoimmune gastritis: pan-atrophic corpus mucosa and normal antral mucosa. Magnifying narrow band imaging was also indicative of autoimmune gastritis: large mucosal atrophy with foveola micromucosal pattern which has a sensitivity of 71% and specificity of 100%.^3)^ The lesion was resected en bloc using endoscopic submucosal dissection technique, which allowed precise pathological evaluation of the margins. Histologically, the tumor exhibited components ranging from moderately to poorly differentiated, with a pale cytoplasm (**Figs. 1A**–**1C**). Immunohistochemistry revealed abundant SALL4-positive cells intermingled with synaptophysin-positive cells, along with scattered alpha-fetoprotein-positive areas (**Figs. 1D** and **1F**). Pathological staging was determined (pT1a(M), ly0, v0, pHM0, pVM0) and indicated complete resection. The specimen showed no evidence of lymphovascular invasion, suggesting the appropriateness of endoscopic surgical management. The background mucosa exhibited features of autoimmune gastritis, including decreased proton pump-positive cells and increased ECL cells (**Figs. 2A**–**2D**), whereas the antrum showed elevated gastrin-positive cells (**Figs. 2E**–**2H**). The patient remains recurrence-free at 12 months.

**Fig. 1:**
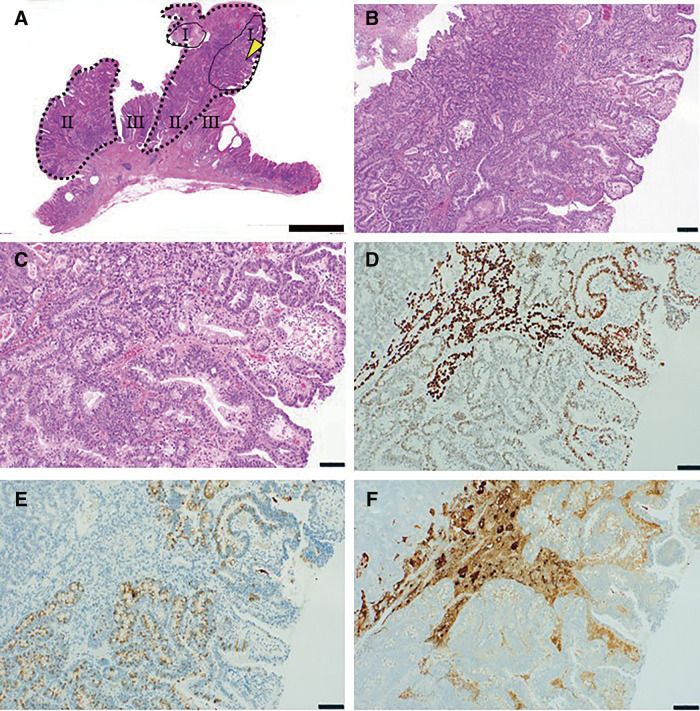
Histopathological and immunohistochemical features of adenocarcinoma. ESD specimen analysis. (**A**) H&E staining showing three distinct areas (I–III) defined by immunophenotype: I) SALL4(+)/Synaptophysin(+), II) SALL4(+)/Synaptophysin(-), III) SALL4(-)/Synaptophysin(-). (**B**, **C**) High-magnification of area I showing tubular proliferation with nuclear atypia. (**D–F**) Immunostaining demonstrating SALL4 (**D**), synaptophysin (**E**), and AFP (**F**) expression patterns. Scale bars: A = 2000 μm, B = 200 μm, C–F = 100 μm.

**Fig. 2:**
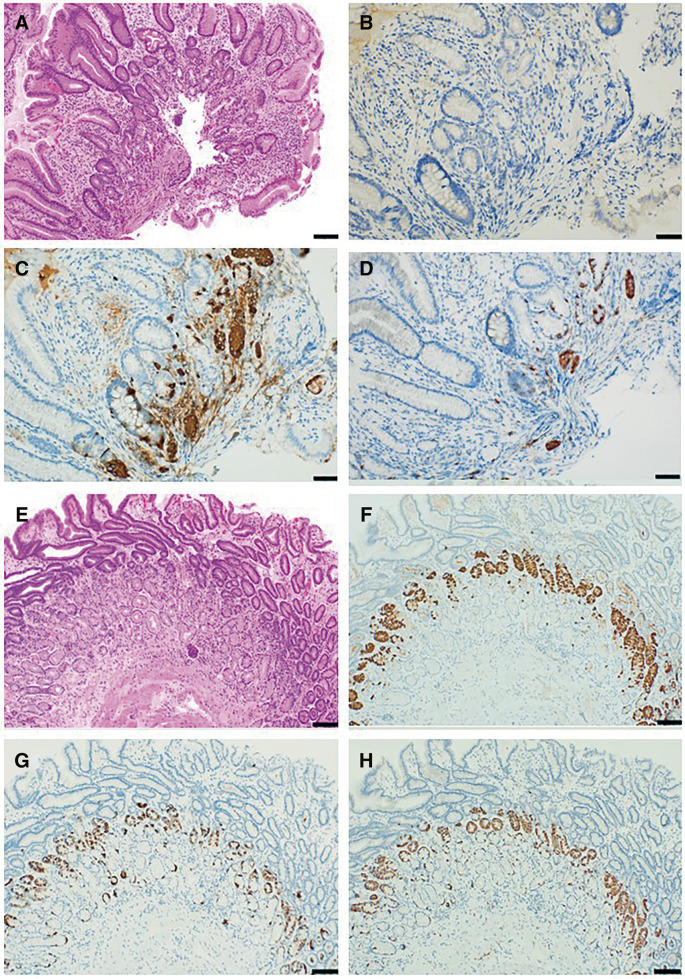
Background gastric mucosa showing autoimmune gastritis. Background mucosa features. (**A–D**) Gastric body: (**A**) H&E staining showing atrophic fundic glands with foveolar hyperplasia, (**B**) loss of proton pump expression, (**C**, **D**) chromogranin- and synaptophysin-positive cell clusters. (**E–H**) Cardiac antrum: (**E**) H&E staining showing preserved pyloric glands, (**F**) increased gastrin-positive cells, (**G**, **H**) increased chromogranin- and synaptophysin-positive cells. Scale bars: A, E–H = 100 μm, B–D = 50 μm.

This case offers novel insights into the relationship between endocrine and embryonic tumors in autoimmune gastritis. Although a single case of ACED associated with autoimmune gastritis has been previously reported,^4)^ to our knowledge, its coexistence with neuroendocrine features has not been documented. This rare case offers insights for both endoscopists and surgeons in determining optimal treatment strategies and understanding the pathogenesis of tumors arising in the context of autoimmune gastritis. Future studies with larger cohorts are needed to elucidate the pathogenesis of mixed-phenotype tumors originating from endocrine cell micronests.

## ACKNOWLEDGMENTS

The authors thank Editage (www.editage.jp) for English language editing.

## DECLARATIONS

### Funding

The authors received no specific funding for this work.

### Authors’ contributions

HS drafted the manuscript;

SI and NU made critical revision;

SM, YA, TM, and RI contributed to data collection; and KH approved the final version of the manuscript.

All authors have read and approved the final manuscript.

### Ethics approval and consent to participate

This case report was approved by the Institutional Review Board at Osaka International Cancer Institute (No. 24026). The patient provided informed consent.

### Consent for publication

The patient gave written informed consent for use of medical information including endoscopic images for this case report. This consent also covers publication.

### Competing interests

The authors declare that they have no competing interests.
